# Regional citrate anticoagulation in hemodialysis: an observational study of safety, efficacy, and effect on calcium balance during routine care

**DOI:** 10.1186/s40697-016-0113-x

**Published:** 2016-04-20

**Authors:** Richard F. Singer, Oliver Williams, Chari Mercado, Bonny Chen, Girish Talaulikar, Giles Walters, Darren M. Roberts

**Affiliations:** The Australian National University, Acton, Australia; The Canberra Hospital, Level 2, Building 15, PO Box 11, Woden, 2606 Australia

**Keywords:** Anticoagulation, Balance, Calcium, Citrate, Flux, Hemodialysis

## Abstract

**Background:**

Regional citrate hemodialysis anticoagulation is used when heparin is contraindicated, but most protocols require large infusions of calcium and frequent intradialytic plasma ionized calcium measurements.

**Objectives:**

The objective of this study was to determine the safety, efficacy, and effect on calcium balance of regional citrate anticoagulation using sparse plasma ionized calcium sampling.

**Design:**

The design of this study was observational.

**Setting:**

The setting of this study was the hospital hemodialysis center.

**Patients:**

The subjects of this study were the hospital hemodialysis patients.

**Measurements:**

Dialysate calcium concentration by atomic absorption spectroscopy and total dialysate weight were used as measurements.

**Methods:**

Regional citrate anticoagulation was introduced using zero calcium dialysate, pre-dialyzer citrate infusion, and post-dialyzer calcium infusion. Infusions were adjusted based on pre- and post-dialyzer calcium measurements obtained at least twice during a 4-h dialysis. The protocol was simplified after the first 357 sessions to dispense with post-dialyzer calcium measurements. Heparin-anticoagulated sessions were performed using acetate-acidified 1.25 mmol/L calcium or citrate-acidified 1.5 mmol/L calcium dialysate. Calcium balance assessment was by complete dialysate recovery. Safety and efficacy were assessed prospectively using a point-of-care database to record ionized calcium and clinical events. Groups were compared using *t* test, ANOVA, Wilcoxon rank sum, or Kruskal-Wallis as appropriate.

**Results:**

Seventy-five patients received regional citrate-anticoagulated dialysis over 1051 dialysis sessions. Of these, 357 dialysis sessions were performed using the original citrate anticoagulation protocol and 694 using the simplified protocol. Dialysis was effective and safe. Only 3 dialyzers clotted; 1 patient suffered symptomatic hypercalcemia and none suffered symptomatic hypocalcemia. Calcium balance was assessed in 15 regional citrate-anticoagulated dialysis sessions and 30 heparin-anticoagulated sessions. The median calcium loss was 0.8 mmol/h dialyzed in both groups (*p* = 0.43), and end of treatment ionized calcium was the same in both groups (1.07 ± 0.04 mmol/L).

**Limitations:**

Our findings for calcium balance, efficacy, and safety are valid only for the protocol studied, which excluded patient with severe liver dysfunction.

**Conclusions:**

Regional citrate dialysis can be performed safely and effectively using a sparse plasma calcium sampling protocol. The calcium balance induced by this protocol is not different to that seen in standard heparin-anticoagulated dialysis, but in the absence of prospective studies, it is unknown whether this is optimal for patient care.

## ABRÉGÉ

**Mise en contexte:**

On recommande l’utilisation de l’anticoagulation régionale au citrate (ARC) d’un circuit de dialyse lorsque l’héparine est contre-indiquée. Toutefois la plupart des protocoles requièrent alors une perfusion de calcium et de fréquentes mesures intradialytiques du taux de calcium ionisé plasmatique.

**Objectifs de l’étude:**

L’étude visait à déterminer l’innocuité l’efficacité et les effets sur l’équilibre calcique de l’ARC à l’aide d’un protocole de prélèvements épars du calcium ionisé plasmatique.

**Type d’étude et participants:**

Il s’agit d’une étude observationnelle menée sur les patients de centres d’hémodialyse en milieu hospitalier.

**Méthodologie:**

L’anticoagulation régionale au citrate implique l’utilisation de trois différentes solutions : un dialysat sans calcium une perfusion de calcium pré-dialyseur ou une perfusion de calcium post-dialyseur. Les perfusions avaient été ajustées selon les taux de calcium mesurés au minimum deux fois pré-dialyseur et post-dialyseur, au cours d’une hémodialyse de quatre heures. Après 357 séances, la mesure du taux de calcium post-dialyseur a été éliminée pour simplifier le protocole. Les séances où l’héparine agissait à titre d’anticoagulant ont été effectuées au moyen de deux différents dialysats : une solution à 1,25 mmol/L de calcium acidifiée à l’acétate et une solution à 1,5 mmol/L de calcium acidifiée au citrate. Le bilan calcique s’est fait en récupérant la totalité du dialysat. L’innocuité et l’efficacité ont été évaluées de façon prospective en consignant les taux de calcium ionisé et les événements cliniques à l’aide des bases de données du lieu de soins. Les groupes de patients ont été comparés en utilisant le test T, l’ANOVA, le test signé des rangs de Wilcoxon ou le test de Kruskal-Wallis selon le cas.

**Résultats:**

Au cours de l’étude 75 patients ont reçu des traitements d’hémodialyse sous anticoagulation régionale au citrate, pour un total de 1051 séances analysées. De celles-ci, 357 séances ont été effectuées en suivant le protocole original d’anticoagulation au citrate, alors que 694 l’ont été en suivant le protocole simplifié. La dialyse s’est avérée sécuritaire et efficace : seuls trois dialyseurs ont coagulé, un seul patient a souffert d’hypercalcémie symptomatique et aucun cas d’hypocalcémie symptomatique n’a été rapporté. Le bilan calcique a été évalué lors de 15 séances d’hémodialyse sous anticoagulation par citrate et de 30 séances utilisant l’héparine. La médiane de perte de calcium se situait à 0,8 mmol/heure de dialyse pour chacun des deux groupes (*p* = 0,43) et le taux de calcium ionisé en fin de séance était le même pour les deux groupes (1,07 ± 0,04 mmol/L).

**Limites de l’étude:**

Ces observations de bilan calcique d’efficacité et d’innocuité ne sont valables que pour le protocole utilisé, excluant ainsi les cas de patients souffrant de dysfonction hépatique grave.

**Conclusions:**

L’anticoagulation régionale au citrate lors de séances d’hémodialyse peut être effectuée à l’aide d’un protocole d’échantillonnage épars du calcium plasmatique. Le bilan calcique établi par ce protocole n’est pas différent de celui observé lors d’une hémodialyse anticoagulée par l’héparine. Toutefois en absence d’études prospectives, nous ignorons si ce protocole est optimal pour prodiguer des soins aux patients.

## What was known before

Regional citrate infusions can successfully anticoagulate a hemodialysis circuit with few immediate complications. However, existing literature mandates frequent plasma ionized calcium measurement to guide changes to calcium infusion rates during therapy.

## What this adds

This study demonstrates that regional citrate anticoagulation performed using a sparse sampling protocol results in a calcium balance equivalent to standard heparin-anticoagulated hemodialysis. Hence, the long-term safety of the procedure is unlikely to be compromised by cumulative calcium overload or deficit. We also demonstrate that the protocol delivers a similar level of immediate safety and efficacy to other published protocols, despite utilizing much less frequent plasma ionized calcium measurement.

## Background

Intermittent hemodialysis is usually performed using a heparin-based anticoagulant, but there are situations where systemic anticoagulation is contraindicated. Unfortunately, anticoagulant-free or reduced dose anticoagulation during hemodialysis increases dialysis circuit clotting [1], leading to interventions such as circuit replacement. Such interventions are expensive and time-consuming. The optimal approach for patients at risk of bleeding is a method that avoids systemic anticoagulation but reliably prevents clotting of the circuit. Regional citrate anticoagulation has been used for this purpose since 1961 [2–5], but uptake has been low probably because plasma calcium monitoring protocols are considered complex. Evidence that simpler protocols can be safe and effective in preventing circuit clotting may improve uptake.

Regional citrate anticoagulation usually involves the use of calcium free dialysate and the infusion of a calcium solution into the dialysis circuit to prevent systemic hypocalcemia [6]. Published protocols are complex when compared to heparin-anticoagulated dialysis because they require frequent intradialytic measurement of plasma ionized calcium [2, 5]. However, even with frequent monitoring, a stable intradialytic [Ca_ion_] does not guarantee a neutral intradialytic calcium balance because it does not take into account calcium mobilization from body stores.

Traditionally, even in bicarbonate-buffered heparin-anticoagulated hemodialysis, the dialysate has contained some acetate to acidify the concentrate. In 2013, dialysates substituting citrate for acetate became available at our institution. The manufacturer (Gambro, Scheelevagen, Sweden), recommended a 1.5 mmol/L dialysate calcium [7–9], rather than our institution’s previous standard 1.25 mmol/L dialysate calcium.

### Study objectives

The primary objective of this observational study was to determine whether regional citrate anticoagulation could be performed safely and effectively by dialysis nurses using protocol-driven changes to infusion rates and sparse plasma calcium sampling. Efficacy and safety were defined as the ability to effectively provide hemodialysis without clotting of the circuit or symptoms related to abnormal plasma calcium. The secondary objectives were to determine the calcium balance during regional citrate anticoagulation and to compare this with the calcium balance during standard heparin-anticoagulated hemodialysis using acetate- or citrate-acidified dialysate.

## Methods

### Study design

This was a prospective, observational study.

### Setting

The setting of this study was the hospital hemodialysis unit.

### Participants

Patients receiving citrate-anticoagulated hemodialysis from October 2011 to June 2015 were enrolled in the regional citrate anticoagulation safety and efficacy arm of the study. Those undergoing regional citrate-anticoagulated hemodialysis in 2012 and those undergoing conventional hemodialysis in 2013 were eligible for enrolment in the calcium balance assessment arm of the study. The use of the citrate-acidified dialysate for heparin-anticoagulated dialysis commenced within the dialysis unit as stocks of acetate-acidified dialysate were exhausted.

Patients assessed for intradialytic calcium balance were selected if they were clinically stable, if they had a well-functioning vascular access, and if their dialysis session occurred when an investigator was available. Participants were not matched and were not randomized to receive a particular dialysate. Dialysis prescription was by the participant’s usual physician, based on their assessment of the optimal therapy. Participants’ dialysis schedule and the availability of an investigator were not expected to vary through the study, so it was anticipated that several patients would be studied with both dialysate compositions. Enrolment continued until 15 sessions in each group (heparin-anticoagulated acetate-acidified dialysate, heparin-anticoagulated citrate-acidified dialysate, and regional citrate-anticoagulated dialysis) were assessed so it was planned to measure the calcium balance in a total of 45 sessions.

### Regional citrate-anticoagulated dialysis procedures

All dialysis treatments utilized a Gambro AK200S machine and Gambro 210H dialyzers (Gambro, Scheelevagen, Sweden) and dialysate part A concentrates manufactured by B. Braun Australia Pty Ltd, Bella Vista, NSW, Australia. Dialysis dose assessment was via automated online recording of ionic clearance. No patients received hemodiafiltration. Dialysate flow was 500 mL/min. Dialysate composition is shown in Table 1.

**Table 1:** Dialysate composition

	Regional citrate anticoagulation	Heparin-anticoagulated citrate dialysate	Heparin-anticoagulated acetate dialysate
Acidification	Acetate 3 mmol/L	Citrate 1 mmol/L	Acetate 3 mmol/L
Na (mmol/L)	140	140	140
Mg (mmol/L)	0.5	0.5	0.5
Dextrose (mmol/L)	5	5	5
Ca (mmol/L)	0	1.5	1.25
K (mmol/L)	1 to 4	1 to 4	1 to 4
HCO_3_^−^(mmol/L)	28	35	35

Anticoagulant Citrate Dextrose, Solution A: Terumo BCT Inc. Belgium (ACD-A) was infused into the “heparin” line, pre-dialyzer, at an initial rate in milliliters per hour of 1.25 times the prescribed blood flow in milliliters per minute. ACD-A contains 22 g/L sodium citrate dihydrate and 8 g/L citric acid monohydrate (113 mmol/L citrate). Calcium chloride of 400 mmol/L was infused into a side port on the “venous” return line at an initial rate in milliliters per hour of 0.143 times the blood flow in milliliters per minute. Therefore, a patient with a prescribed blood flow of 300 mL/min would be commenced at an ACD-A infusion of 375 mL/h (42.3 mmol citrate/h) and a calcium infusion of 17.2 mmol/h.

Plasma total calcium, alanine transferase (ALT), gamma glutamyl transferase (GGT), and bilirubin concentrations were measured prior to the initial citrate-anticoagulated dialysis session for each patient and then at least monthly. During the first 357 hemodialysis sessions, both systemic and post-dialyzer [Ca_ion_] were measured 30 min after commencing dialysis, at completion of dialysis, and during dialysis at times determined by [Ca_ion_] results (Table 2). Patients with abnormal liver function tests (LFT) and abnormal plasma total calcium were not excluded from receiving regional citrate anticoagulation during the first 357 sessions, but such abnormalities were listed as a “caution.”

**Table 2:** Calcium and ACD-A infusion adjustments: original protocol

	Intradialytic ionized calcium (mmol/L)				
	Post-dialyzer ≥ 0.27	Systemic <0.81	Systemic 0.81 ≤ 0.95	Systemic 0.96 ≤ 1.32	Systemic >1.32
Action	Increase ACD-A infusion by 25 %	Increase Ca infusion by 50 %	Increase Ca infusion by 25 %	No action	Reduce calcium infusion by 25 %
Repeat [Ca_ion_] required in	30 min	30 min	60 min	At the completion of dialysis	30 min

A review in December 2012 found that many ionized calcium assessments were performed in response to an elevated post-dialyzer [Ca_ion_] rather than to out of range systemic [Ca_ion_] and that clinical staff were uncertain how to respond to abnormal LFT. The protocol was then modified to dispense with the measurement of post-dialyzer calcium, to mandate an increased frequency of calcium measurements in patients with mildly deranged LFT (Table 3), and to exclude those with severe liver dysfunction from regional citrate anticoagulation. Concurrent with this change, a database was purchased that allowed automated importation of laboratory test results. Mild derangement of liver function was defined as a GGT or ALT concentration greater than the laboratory reference range or a bilirubin >30 μmol/L. These patients were required to have at least hourly assessment of systemic ionized calcium during hemodialysis. Severely deranged LFT were defined as a GGT >600 U/L, an ALT >400 U/L, or a bilirubin >45 μmol/L.

**Table 3:** Calcium infusion adjustments: modified protocol

	Systemic Intradialytic ionized calcium (mmol/L)			
	Systemic <0.81	Systemic 0.81 ≤ 0.95	Systemic 0.96 ≤ 1.32	Systemic >1.32
Action	Increase Ca infusion by 50 %	Increase Ca infusion by 25 %	No action	Reduce calcium infusion by 25 %
Repeat [Ca_ion_] required in	30 min	30 min	2 h	30 min

Adverse reactions, dialyzer clotting and [Ca_ion_] results during citrate dialysis were recorded in real time on a Microsoft Access database developed for this purpose. The database provided the nurses performing dialysis with instructions for altering infusion rates, based on the inputted ionized calcium results. If the database was unavailable due to network problems, nurses were instructed to record results on the clinical dialysis record and to use printed tables to calculate any required changes to infusion rates. These records were entered into the Access database retrospectively. Dialyzer clotting was determined by visual inspection of the dialyzer by the dialysis nurse on a 4 category scale (clear dialyzer, mild streaking, moderate clotting, or severe clotting).

### Heparin-anticoagulated dialysis procedure

Hemodialysis was performed using Gambro machines (AK200S), dialyzers (210H or 170H), and concentrates (for composition see Table 1). Dialysate flow was 500 mL/min. No patients received hemodiafiltration.

### Sampling

At the commencement of the dialysis, but prior to the commencement of the blood pump, a sample of dialysate was collected directly from the dialysate effluent tube to measure the calcium concentration. Urea and sodium were also measured, to confirm correct timing of the dialysate samples. The dialysate effluent tube was then positioned over a clean plastic vessel and the blood pump started. The weight of dialysate collected was measured using calibrated scales, and volume was calculated based on a mean spent dialysate density of 1.02 kg/L. This density figure was obtained from four random samples measured in triplicate. At the completion of the dialysis session, the effluent was mixed for 3 min in an alternating clockwise and anticlockwise direction, following which a sample was taken for quantification of spent dialysate calcium, urea, and sodium. Intradialytic blood samples were taken for [Ca_ion_] measurement in the regional citrate-anticoagulated participants, whereas in heparin-anticoagulated participants, sampling was at the commencement and completion of dialysis.

### Laboratory analysis

All laboratories that performed sample analysis for this study were accredited by the National Association of Testing Authorities, Australia.

Total dialysate calcium was initially analyzed using the Architect (Arsenazo III) platform; however, some spent dialysate concentrations were below the detection threshold and the assay was changed to an inductively coupled plasma atomic emission spectroscopy (ICPAES) method [10]. The analytical laboratory reported a precision of 5 % and a lower reporting limit of 0.00075 mmol/L. All reported dialysate calcium results are based solely on the ICPAES assay.

Blood and dialysate ionized calcium were analyzed using an IL GEM Premier 4000 blood gas analyzer for which the company reported a precision in blood samples of 0.71 % at 1.59 mmo/L and 1.1 % at 0.82 mmol/L. The reference range provided by the laboratory for this assay was 1.12 to 1.32 mmol/L.

Dialysate citrate was measured by the UV-method using commercial kits produced by Boehringer Mannheim. The laboratory reported a precision of 2 % for this test.

### Calculation of intradialytic calcium balance and fractional citrate removal

All citrate and calcium balance assessments during regional citrate anticoagulation were performed under the initial (pre- and post-dialyzer) calcium sampling protocol. Regional citrate-anticoagulated intradialytic calcium balance and citrate removal were calculated according to the equations

$$ \mathrm{intradialytic}\ \mathrm{C}\mathrm{a}\ \mathrm{loss} = \left(\left({\left[\mathrm{C}\mathrm{a}\right]}_{\mathrm{outlet}}-{\left[\mathrm{C}\mathrm{a}\right]}_{\mathrm{inlet}}\right)\times 0.98\times {D}_{\mathrm{weight}}\right)-\left({\left[\mathrm{C}\mathrm{a}\right]}_{\mathrm{infused}}\times \mathrm{infused}\ \mathrm{volume}\right) $$

$$ {\mathrm{Citrate}}_{\mathrm{fractional}\ \mathrm{removal}}=1-\frac{\left({\left[\mathrm{Citrate}\right]}_{\mathrm{infused}}\times \mathrm{Infused}\ \mathrm{volume}\left)-\right({\left[\mathrm{Citrate}\right]}_{\mathrm{outlet}}\times 0.98\times {D}_{\mathrm{weight}}\right)}{{\left[\mathrm{Citrate}\right]}_{\mathrm{infused}}\times \mathrm{infused}\ \mathrm{volume}}\Big) $$

Heparin-anticoagulated intradialytic calcium balance was calculated according to the equation

$$ \mathrm{intradialytic}\ \mathrm{C}\mathrm{a}\ \mathrm{loss} = \left({\left[\mathrm{C}\mathrm{a}\right]}_{\mathrm{outlet}}-{\left[\mathrm{C}\mathrm{a}\right]}_{\mathrm{inlet}}\right)0.98\times {D}_{\mathrm{weight}} $$

Where [Ca]_outlet_ is the mixed total dialysate effluent calcium concentration, [Ca]_inlet_ is the total dialysate calcium concentration pre-dialyzer, [Ca]_weight_ is the total dialysate effluent weight in kilograms, and [Ca]_infused_ is the concentration of calcium infused post-dialyzer.

Since dialysis session length varied, balance was calculated as a loss per hour of dialysis. The calcium balance reported includes losses related to ultrafiltration. No patient had significant residual renal function. As this study was an assessment of extracorporeal therapy on calcium balance, the effects of food consumed or urine passed during dialysis were not relevant.

### Ethics statement

The need for regional citrate-anticoagulated dialysis or standard heparin-anticoagulated dialysis was determined by physician assessment of clinical need. Calcium balance assessment did not involve any contact with the patients, or a change to their care, so patients were not considered to be participating in a clinical trial, and written consent was not considered necessary. This decision was reviewed and endorsed by the Australian Capital Territory Human Research Ethics Committee. During the second phase of the study (in heparin-anticoagulated patients), the Ethics Committee requested that written consent be obtained from participants and all subjects provided this consent prior to participation.

### Statistics

Normally distributed data are expressed as mean ± standard deviation with differences tested using *t* test, paired *t* test, or ANOVA as appropriate. Equality of variance was confirmed by Levene’s test and parametric distribution by Shapiro-Wilk test. Non-normally distributed data are expressed as medians with range or interquartile range (IQR) with differences tested using Wilcoxon or Kruskal-Wallis tests as appropriate. Differences in categorical variables were tested with Fisher’s exact test, and correlations were performed using Spearman rank correlation (*r*_s_). Statistical significance was set at a two-tailed *p* value of <0.05. Power analysis, for the minimum detectable difference in dialysis calcium balance could not be performed, as there was no suitable published data on which to base such an analysis. Each dialysis session was considered to be an independent observation. This was on the basis that an approximately neutral balance was anticipated and that the calcium balance in one session was therefore unlikely to have an effect on subsequent sessions.

## Results

Patient and dialysis characteristics for participants involved in calcium balance assessment, and calcium data are summarized in Table 4. A total of 75 patients received regional citrate-anticoagulated dialysis. The indication was heparin allergy in 2 patients and high bleeding risk in the remainder. There were 357 sessions performed using the original citrate anticoagulation protocol and 694 sessions using the modified protocol (systemic plasma calcium sampling only). Three or more blood draws for systemic [Ca_ion_] assessment were needed in 31 % of sessions performed under the original protocol and 15 % under the modified protocol (*p* < 0.0005). Treatment complications during regional citrate anticoagulation were rare and are summarized in Table 5. There were no episodes of symptomatic hypocalcemia and one episode of symptomatic hypercalcemia (vomiting, due to the patient mistakenly receiving 4 times the prescribed dose of intravenous calcium). Hypercalcemic episodes in the modified protocol were associated with higher hemoglobin concentrations (*p* = 0.0001). The median hemoglobin was 152 (IQR 142 to 160 g/L) in sessions complicated by hypercalcemia and 116 (IQR 103 to 130 g/L) in sessions where hypercalcemia did not occur. One patient underwent 80 sessions under the modified citrate anticoagulation protocol and 7 of these sessions were complicated by hypercalcemia. The median hemoglobin of this patient in sessions complicated by hypercalcemia was 158 g/L, compared to 127 g/L in sessions where hypercalcemia did not occur (*p* = 0.005). Insufficient data on hemoglobin concentrations of patients dialyzed under the original protocol were available for analysis. No patients receiving heparin-anticoagulated dialysis as part of the calcium balance study experienced an adverse event.

**Table 4:** Calcium balance assessment: demographic and dialysis characteristics

	Heparin-anticoagulated acetate-acidified dialysate (*n* = 15)	Heparin-anticoagulated citrate-acidified dialysate (*n* = 15)	Regional citrate-anticoagulated dialysis Sessions (*n* = 15)	*p* value
Age (years)	76 (range 67–90)	77 (range 55–86)	84 (range 59–87)	0.62
Sex	4 male, 5 female	4 male, 5 female	5 male, 1 female	0.3
Median dialysis sessions per patient	1 (range 1–3)	1 (range 1–3)	1 (1–5)	
Gambro Dialyzer 210H	13	13	15	
Gambro Dialyzer 170H	2	2	0	
Blood flow (mL/min)	350 (IQR 305 to 350)	330 (IQR 310 to 350)	300 (IQR 300 to 300)	0.0008
Kt/V per session	1.24 (IQR 1.2 to 1.37)	1.42 (IQR 1.26 to 1.54)	1.23 (IQR 1.06 to 1.28)	0.01
Dialysis session duration (minutes)	240 (range 240 to 270)	240 (range 240 to 270)	240 (range 210 to 300)	1
Prescribed net ultrafiltration (L/h dialyzed)^a^	0.66 ± 0.21	0.71 ± 0.23	0.59 ± 0.24	0.4
Calcium infused (mmol/h dialyzed)	0	0	17.2 (range 11.6 to 20)	
Spent dialysate volume (L)	124.0 (IQR 122.7 to 125.0)	124.0 (IQR 122.4 to 124.8)	131.0 (127 to 148.7)	0.002
Dialysate [Ca_ion_] prior to commencement of dialysis in mmol/L	1.04 (range 1.0 to 1.09)	0.85 (range 0.83 to 1.05)		<0.00005
Initial dialysate total Ca (mmol/L)	1.22 ± 0.03	1.43 ± 0.07	0.01 ± 0.004	<0.00005
Spent dialysate total Ca (mmol/L)	1.25 ± 0.04	1.46 ± 0.05	0.55 ± 0.07	<0.00005
Start of session plasma [Ca_ion_] (mmol/L)	1.12 ± 0.09	1.12 ± 0.1		0.91
End of session plasma [Ca_ion_] (mmol/L)	1.09 ± 0.04	1.06 ± 0.04	1.06 ± 0.05	0.21

^a^Data on net ultrafiltration was missing for 1 session in each heparin-anticoagulated group

**Table 5:** Efficacy and safety of regional citrate anticoagulation

	Original regional citrate protocol (*n* = 357)	Modified regional citrate protocol (*n* = 694)	*p* value
Dialyzer clotting	1 (0.3 %) severe	2 (0.3 %) moderate streakiness	1
Systemic [Ca_ion_] <0.81 mmol/L	2 (0.6 %)	1 (0.1 %)	0.27
Systemic [Ca_ion_ ] <0.96 mmol/L	35 (9.8 %)	26 (3.7 %)	<0.0005
Systemic [Ca_ion_] >1.32 mmol/L	2 (0.6 %)	12 (1.7 %)	0.16

In 591 sessions which used the modified protocol, 30 min post-commencement intradialytic hemoglobin data was available. There was a linear correlation between this hemoglobin and systemic [Ca_ion_] at the same time point (*r* = 0.41, *p* < 0.0005).

### Calcium balance and citrate extraction in regional citrate-anticoagulated sessions

Dialysis effluent was collected for 27 dialysis sessions in 8 participants receiving regional citrate anticoagulation. Arteriovenous fistula were used in 26 of these sessions. Due to sample processing errors, 5 effluent citrate and 3 effluent calcium measurements were unusable. A further 9 calcium measurements were discarded as they were obtained using the Architect platform. Therefore, 22 sessions were available for assessment of citrate extraction and 15 sessions (in 6 patients) for assessment of calcium balance.

No participant recorded a systemic [Ca_ion_] below 1 mmol/L or required an adjustment of the calcium infusion during dialysate collection but seven required a 25 % increase in the citrate infusion rate due to an elevated post-dialyzer [Ca_ion_] 30 min into dialysis. Higher citrate infusions did not consistently alter the subsequent systemic [Ca_ion_]. Following a rise in citrate dose, systemic [Ca_ion_] rose in two sessions but fell in four sessions (mean fall 0.01 mmol/L, *p* = 0.42).

A positive intradialytic calcium balance occurred in two sessions, with the remainder leading to a net calcium loss. The mean calcium loss was 1.15 ± 1.11 mmol (median 0.80, IQR 0.30 to 1.9 mmol/L) per hour of dialysis. The fractional removal of infused citrate during dialysis was 0.76 (IQR 0.74 to 0.78).

### Comparison to calcium balance in heparin-anticoagulated dialysis sessions

Intradialytic calcium balance was assessed in 30 heparin-anticoagulated dialysis sessions: 15 using citrate-acidified dialysate and 15 using acetate-acidified dialysate in a total of 12 patients. All patients utilized an arteriovenous fistula for dialysis. Six patients underwent measurement of calcium balance in both citrate- and acetate-acidified dialysate sessions.

Systemic [Ca_ion_] fell during dialysis in both heparin-anticoagulated groups by a mean of 0.04 mmol/L in the acetate-acidified (*p* = 0.03) and 0.06 mmol/L in the citrate-acidified sessions (*p* = 0.03), with no between group difference (*p* = 0.45). The mean post-dialysis [Ca_ion_] was 1.07 ± 0.04 mmol/L across the 45 sessions and was not statistically different between dialysate groups (Table 4).

**Fig. 1:**
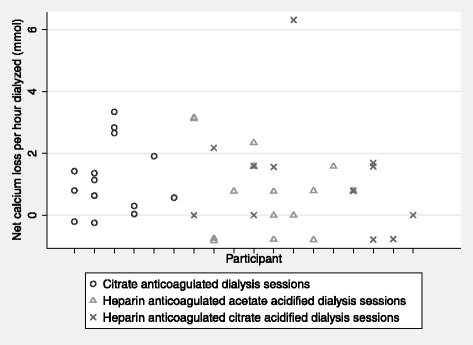
Participant calcium loss in heparin- and citrate-anticoagulated dialysis

The net median calcium loss in the heparin-anticoagulated groups was 0.8 (IQR 0 to 1.6 mmol/h). There was no difference in calcium losses between the heparin-anticoagulated dialysate groups (*p* = 0.71) and no difference between those groups combined and the citrate-anticoagulated sessions (*p* = 0.43). No trend was apparent in sessional calcium losses between the same heparin-anticoagulated patients utilizing different dialysates, although this could not be formally tested due to the small numbers involved. Per participant calcium balance data is summarized in Fig. 1.

**Fig. 2:**
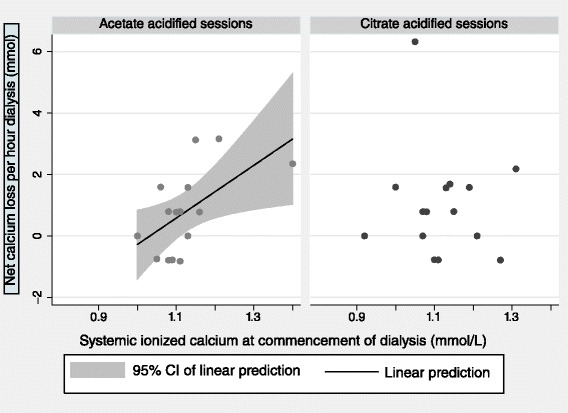
Calcium loss vs systemic ionized calcium at the commencement of dialysis

The plasma [Ca_ion_] at commencement of dialysis and the net hourly calcium loss were correlated in the heparin-anticoagulated acetate-acidified sessions (*r*_s_ 0.54, *p* = 0.04) but not in the citrate-acidified sessions (*r*_s_ = −0.03). This is shown graphically in Fig. 2.

## Discussion

The regional citrate anticoagulation algorithm studied provided effective anticoagulation in 99.7 % of sessions and resulted in similar end of dialysis systemic [Ca_ion_] to those seen in heparin-anticoagulated dialysis. In the modified protocol, 85 % of sessions required only two blood draws for [Ca_ion_] measurement over a 4-h dialysis, which is half the number of tests mandated in previously published algorithms [2, 5]. Dialysis nurse staff satisfaction with the protocol was not formally measured but appeared to be high, with nurses frequently requesting that physicians prescribe regional citrate anticoagulation, (in preference to heparin-free, saline flush dialysis) as they felt it was less laborious. Adverse events were rare, with only one symptomatic hypercalcemia and no symptomatic hypocalcemia. An increase in the calcium dose at commencement of dialysis would likely have reduced the number of episodes of asymptomatic hypocalcemia but would probably have caused more hypercalcemic episodes, particularly in those with a higher hemoglobin.

Hemoglobin is relevant to regional citrate algorithms because erythrocytes are impermeable to citrate [11] so the citrate and calcium doses necessary for effective anticoagulation and safe dialysis are more closely related to plasma flow rather than blood flow. We did not correct dosing for hemoglobin as this would have increased the complexity of the algorithm, and we were concerned at the risk of additional administration errors. The numerically greater incidence of hypercalcemia in the modified protocol was probably related to higher hemoglobin concentrations but lack of adequate hemoglobin data during use of the original protocol prevented further analysis of this.

We employed a robust method (complete spent dialysate collection) to assess calcium balance and found that calcium balance was not different between heparin- and citrate-anticoagulated sessions. The heparin-anticoagulated sessions acidified with acetate or citrate resulted in equivalent intradialytic balance despite containing different calcium concentrations.

Kozik-Jaromin investigated regional citrate-anticoagulated dialysis calcium using an intermittent blood sampling technique and found a similar fractional citrate removal to our study but a *positive* mean calcium balance of approximately 1 mmol/h dialyzed [12]. The positive balance is almost certainly due to the use of a 7 % higher initial calcium infusion rate, compared to ours. Interestingly, they appeared to have a higher complication rate with 2 of 15 sessions studied being affected by hypo- or hypercalcemia. Calcium balance probably depends mostly on the calcium infusion rate because citrate, unlike calcium, is infused pre-dialyzer and mostly dialyzed off. Our algorithm used a similar initial calcium substitution rate to Apsner’s [2] and a 23 % higher rate to that used by Wright [5].

KDIGO guidelines suggest most patients should be dialyzed using a 1.25 to 1.5 mmol/L calcium dialysate, to achieve approximately neutral calcium balance [13]. The citrate algorithm assessed in our study led to a small net calcium loss that was equivalent to the losses seen in heparin-anticoagulated sessions. Whether this results in deleterious effects on bone, or perhaps in beneficial effects on vascular calcification, is unknown and could only be determined with a much larger, controlled, longitudinal study.

Our data are presented inclusive of losses due to ultrafiltration and exclusive of both dietary calcium absorption and urinary calcium losses. Ultrafiltration contributes to calcium flux, but measurement is impractical during concurrent dialysis. At approximately 0.6 L/h, net ultrafiltration likely accounts for much of the negative calcium balance seen. Performing the study in the absence of ultrafiltration would have allowed the diffusive calcium balance to be determined but is not how patients are dialyzed in clinical practice and is not the “real world” calcium balance experienced by the patient. Non-dialysis calcium losses and gut absorption were not relevant to this study as it was a study into extracorporeal losses induced by dialysis and not the overall calcium balance of patients.

Similar to a previous study [14], we found that the pre-dialysis [Ca_ion_] correlated with intradialytic calcium balance in the acetate dialysate sessions. This is consistent with studies indicating that the plasma [Ca_ion_] is the dialyzable calcium fraction, at least in the absence of citrate [15, 16]. Plasma citrate rises during citrate-acidified dialysate sessions [17], chelates calcium, and causes a rise in the concentration of dialyzable, non-ionized, calcium-citrate chelates [18]. Without a concomitant increase in dialysate calcium concentration, the effect of adding citrate to dialysate is an increase in calcium losses during dialysis. We found that pre-dialysis plasma [Ca_ion_] in citrate-acidified dialysis sessions did not correlate with intradialytic calcium balance. This is probably because individuals' homeostatic response to increased blood citrate varies and negates the correlation that would otherwise be seen.

## Limitations

Our findings for calcium balance, efficacy, and safety are valid only for the protocol studied and cannot necessarily be extrapolated to use in patients with more severe liver disease, markedly different hemoglobin concentrations or an alternative protocol. For example, a sparse sampling protocol may not detect incipient citrate toxicity at an early stage in those with severe liver disease.

## Conclusions

Regional citrate anticoagulation using a simple, sparse sampling algorithm allows effective hemodialysis with a low rate of symptomatic adverse events and an equivalent calcium balance to standard heparin anticoagulation. Although the citrate-acidified dialysate utilized in heparin-anticoagulated sessions contained a 20 % greater calcium concentration than the acetate-acidified dialysate, it resulted in the same calcium balance. This suggests that future dialysate guidelines should consider the effects of dialysate acid when recommending a dialysate calcium concentration.

## Abbreviations

[Ca_ion_]: ionized calcium concentration in mmol/L
ALT: alanine transferase
GGT: gamma glutamyl transferase
LFT: liver function tests
